# Global gene expression in endometrium of high and low fertility heifers during the mid-luteal phase of the estrous cycle

**DOI:** 10.1186/1471-2164-15-234

**Published:** 2014-03-26

**Authors:** Aideen P Killeen, Dermot G Morris, David A Kenny, Michael P Mullen, Michael G Diskin, Sinéad M Waters

**Affiliations:** Teagasc, Animal and Bioscience Research Department, Animal and Grassland Research and Innovation Centre, Grange, Dunsany, County Meath, Ireland; UCD School of Agriculture, Food Science and Veterinary Medicine, College of Life Sciences, University College Dublin, Belfield, Dublin 4, Ireland; Teagasc, Animal and Bioscience Research Department, Animal and Grassland Research and Innovation Centre, Mellows Campus, Athenry, County Galway, Ireland

## Abstract

**Background:**

In both beef and dairy cattle, the majority of early embryo loss occurs within the first 14 days following insemination. During this time-period, embryos are completely dependent on their maternal uterine environment for development, growth and ultimately survival, therefore an optimum uterine environment is critical to their survival. The objective of this study was to investigate whether differences in endometrial gene expression during the mid-luteal phase of the estrous cycle exist between crossbred beef heifers ranked as either high (HF) or low fertility (LF) (following four rounds of artificial insemination (AI)) using the Affymetrix® 23 K Bovine Gene Chip.

**Results:**

Conception rates for each of the four rounds of AI were within a normal range: 70–73.3%. Microarray analysis of endometrial tissue collected on day 7 of the estrous cycle detected 419 differentially expressed genes (DEG) between HF (n = 6) and LF (n = 6) animals. The main gene pathways affected were, cellular growth and proliferation, angiogenesis, lipid metabolism, cellular and tissue morphology and development, inflammation and metabolic exchange. DEG included, *FST*, *SLC45A2*, *MMP19*, *FADS1* and *GALNT6*.

**Conclusions:**

This study highlights, some of the molecular mechanisms potentially controlling uterine endometrial function during the mid-luteal phase of the estrous cycle, which may contribute to uterine endometrial mediated impaired fertility in cattle. Differentially expressed genes are potential candidate genes for the identification of genetic variation influencing cow fertility, which may be incorporated into future breeding programmes.

**Electronic supplementary material:**

The online version of this article (doi:10.1186/1471-2164-15-234) contains supplementary material, which is available to authorized users.

## Background

The failure of breeding females to become pregnant, in both dairy and beef cattle production systems, directly impacts the economic viability of these enterprises, and ultimately hinders genetic progress. Significant decreases in dairy cow fertility, ranging from 0.45% to 1% per annum, have been reported in cattle populations across the globe [1–3]. Following insemination the greatest increment of cow reproductive wastage occurs in the form of early embryo mortality with approximately 80% of this occurring within 14–16 days [4–6]. More specifically, previous studies have highlighted that the majority of early embryo loss typically commences around the mid-luteal phase of an estrous cycle i.e. day 7 of pregnancy [7, 8] concurrent with the critical blastulation stage of embryo development [9].

There is evidence of repeatable differences between cows in their ability to become pregnant. McMillan [10] reported a 65% difference in pregnancy rate at 60 days of gestation, following 6 consecutive *in vitro* embryo transfer events, between two groups of cows. Differences in follicle wave dynamics, duration of estrus, site of ovulation, or subsequent progesterone profiles were not found to contribute to the observed difference in pregnancy rate. Indeed, the authors suggested that “uterine” rather than “ovarian” factors may be responsible for the variation observed. This uterine effect was also hypothesized in similar studies examining phenotypic differences between high and low fertility animals [11–13]. Furthermore, data from our laboratory suggest a repeatability estimate of 0.18 for embryo survival in beef heifers [13] and heritability estimates for conception rate have been reported to exceed 0.20 [14, 15].

The prerequisites to the establishment and maintenance of a successful pregnancy include a viable embryo, an appropriate steroidal environment and an optimally functioning and receptive endometrium [16–18]. The endometrium plays a pivotal role in orchestrating the events that lead to fertilization, implantation and pregnancy. Throughout the estrous cycle and pregnancy, the endometrium is subjected to a host of functional and morphological changes, regulated by the hormones progesterone, estradiol and oxytocin [19]. The endometrium also functions to secrete a multitude of growth factors, proteins and cytokines, all of which constitute the histotroph, an important source of energy and nutrition to a growing embryo *in vivo*[20–22].

Using conventional candidate approaches many studies have examined bovine endometrial gene expression under various conditions; during early pregnancy in animals that produced viable and non-viable embryos [23], in pregnant and cycling animals with artificially induced high, and normal systemic progesterone concentrations [24–26] and during the various phases of the estrous cycle [27]. Furthermore global endometrial gene expression analyses have been conducted and include comparisons between cycling and pregnant animals [28, 29], fertile and sub-fertile animal strains [30, 31], progesterone supplementation treatments [32], and specific estrous cycle phases [33, 34]. Despite these efforts, endometrial gene expression of animals characterized as either high or low fertility has not been investigated. Given the critical importance of day 7 [7, 8], we hypothesise that uterine endometrial gene expression patterns will be different between high and low fertility heifers on day 7 of the estrous cycle. Thus, the objective of this study was to characterize differential gene expression profiles in endometrial tissue harvested on day 7 of the estrous cycle from heifers ranked as either HF or LF fertility based on four successive inseminations and pregnancy diagnoses. Intercaruncular endometrial tissue was examined due to the fact that caruncular endometrium lacks uterine glands which are essential to the exchange, transport and secretion of pertinent metabolites which constitute the uterine histoptroph and are required to support pregnancy [35, 36].

## Methods

### Ethics statement

All experimental procedures involving heifers were licensed by the Department of Health and Children, Ireland (licence number B100/846). Protocols were in accordance with the Cruelty to Animals Act (Ireland 1876, as amended by European Communities regulations 2002 and 2005) and the European Community Directive 86/609/EC and were sanctioned by the Institutional Animal Research Ethics Committee.

### Animal model

Estrous cycles of reproductively normal nulliparous crossbred beef heifers (*Bos taurus* n = 120) were synchronized using two intramuscular administrations of 500 μg of the prostaglandin F_2α_ analogue (PG), cloprostenol (Estrumate®, Schering-Plough Ltd., Shire Park, Welwyn Garden City, Hertfordshire, UK). Animals were visually observed for signs of estrous activity 3- to 5- times daily as described by Lynch et al. [7]. Only heifers observed to be in standing estrus were inseminated 6–18 hrs after onset of heat [37]. Inseminations were carried out artificially by one trained technician. Heifers were given a single insemination of frozen-thawed semen, collected from a single ejaculate of one high fertility bull. Sire breed was Limousin and named Bolide (FL17). At the time of the 1st insemination, heifers were on average 20 months of age and weighed 440 ± 9.0 kg (Mean ± SEM).

Using an Aloka SSD-500 V ultrasound scanner, fitted with a 7.5 MHZ transducer (Aloka Co. Ltd., Tokyo, Japan), pregnancy was diagnosed 28 days after insemination using the criteria set out by Kastelic et al. [38]. Following diagnosis, all pregnant heifers received PG on day 28 to induce embryo loss. Six weeks after induced embryo loss all heifers were subjected to estrous reprogramming using a two-injection PG-regimen (11 days apart), inseminated and pregnancy scanned as described above.

For the purpose of establishing an accurate high *versus* low heifer fertility model, this schedule was followed for a further two occasions. Thus, following four inseminations, animals that established a pregnancy on all four occasions were categorized as “HF” heifers while those achieving pregnancy on only one occasion were categorized as “LF” heifers. To eliminate the possibility of a physical or anatomical abnormality that may have impeded heifers from becoming pregnant, animals with zero recorded pregnancies were omitted from the study.

**Figure 1:**
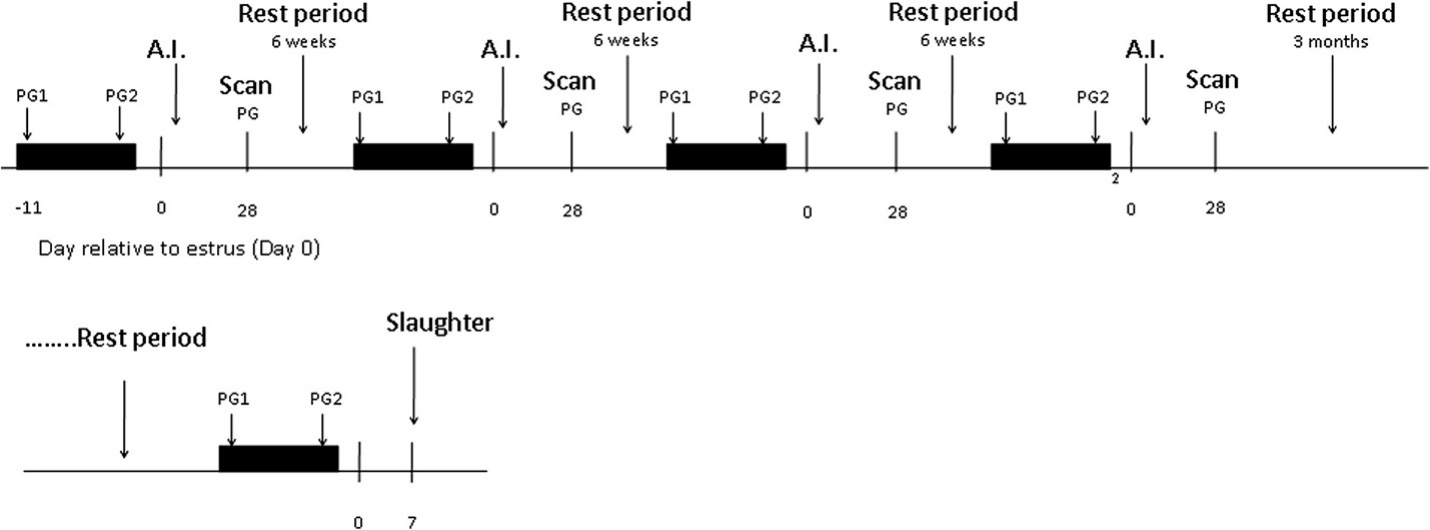
**Experimental design timeline.**

After the fourth insemination, and subsequent pregnancy diagnosis, pregnant heifers were returned to estrous. Approximately three months later, estrous cycles of animals were synchronized again in preparation for endometrial harvesting on D7. Figure 1 illustrates the timeline of events during the experimental period.

Throughout the experimental period, animals had *ad libitum* access to grass silage supplemented with 2 kg of concentrates per heifer per day. Heifers were housed on concrete slats in groups of 15, at 2.5 m^2^ per heifer, for the duration of the study (15 months). Slaughter liveweight averaged 625 kg, with BCS of 4.0. Heifers were gaining weight during the course of inseminations in the region of 0.60 kg/day.

### Tissue sampling

Animals from HF (n = 6) and LF groups (n = 6) were slaughtered on D7 in a licensed abattoir (KEPAK, Athleague, Co. Roscommon, Ireland). Following slaughter the reproductive tract and ovaries were checked for gross abnormalities but none were recorded. Uterine tissues were opened longitudinally along the mesenteric border. Intercaruncular endometrial cross-sections approximately 4 sq cm, and weighing 2.5 g, were harvested from the animals by peeling from the underlying uterine myometrium from the middle-third of the uterine horn ipsilateral to the corpus luteum (CL) within 20 min of slaughter.

Samples were washed in sterile PBS, and stored in RNA*later*® at 4°C for 24 h before being transferred for long-term storage at −20°C. All surgical instruments used for tissue collection were sterilized and treated with RNA Zap (Ambion, Applera Ireland, Dublin, Ireland). In addition, on the day of slaughter CL diameter for each heifer was determined using vernier calipers.

### Blood sampling

Heifers were blood sampled via jugular venipuncture for subsequent measurement of progesterone at 0900 and 2100 h commencing 24 h after PG for a cycle length. All blood samples were collected into 10 ml ethylenediamine tetraacetic acid (EDTA) heparinized Vacutainers (Becton Dickson Vacutainer Systems, Plymouth, UK). Samples were held in iced water until centrifuged at 1500 × *g* at 4°C for 15 mins after which plasma was extracted and stored in sterile 7 ml vials at −20°C until assayed.

### Progesterone assays

Progesterone profiles for each of the six heifers within HF and LF groups were established. Concentration of progesterone was measured in plasma as the mean of the two samples taken on each cycle day of the previous cycle and on 7 days prior to slaughter using the Coat-a-Count assay procedure (Coat-a-Count Diagnostic Products Corporation, Los Angeles, CA, USA) with each sample tested in duplicate. The inter-assay and intra-assay coefficients of variation for low, medium and high control samples were 17.4% and 4.4%, 5.6% and 28.4%, and 4.2% and 4.9% with mean concentrations of 0.24, 2.54 and 7.21 ng/mL, respectively. The minimum detectable limit for this assay was 0.06 ng/mL.

### RNA extraction and quality analysis

Total RNA was prepared from 100–200 mg of endometrial tissue using the TRIzol reagent (Sigma-Aldrich Ireland Ltd., Dublin, Ireland). Tissue samples were homogenized in 3 ml of TRIzol reagent and chloroform, and subsequently precipitated using isopropanol (Sigma-Aldrich Ireland Ltd., Dublin, Ireland). RNA samples were stored at −80°C. Samples of RNA, (20 μg), were purified and treated for contaminating genomic DNA using RNeasy clean-up kits in accordance with manufacturer’s guidelines supplied (QIAGEN, Crawley, West Sussex, UK). This protocol included an on-column DNase treatment step. RNA quality and quantity were assessed using automated capillary gel electrophoresis on a Bioanalyzer 2100 with RNA 6000 Nano Lab-chips according to manufacturer’s instructions (Agilent Technologies Ireland, Dublin, Ireland). Absorbance ratios (28S/18S) and RNA integrity values recorded for all RNA samples extracted post clean-up ranged between 1.8 and 2.0, and 7.5 and 9.8, respectively.

### Microarray hybridization

Gene expression was determined using a 24,027 probe set bovine oligonucleotide array (Affymetrix®), representing ~23,000 bovine transcripts based on the original mapping using Unigene build 57 (March 24, 2004). RNA from each heifer was hybridized to a separate array. All 12 RNA samples were hybridized and scanned by the German Resource Centre for Genomic Research (RZPD), Germany, according to the manufacturer’s instructions.

### Microarray analysis

All microarray analyses including preprocessing, normalization and statistical analysis were carried out using R (R, 2007) version 2.6 and Bioconductor [39] version 2.1 as previously described by [40]. Data were quality assessed before and after normalization using a number of in-built quality control methods implemented in the Bioconductor affycoretools and associated packages to identify problems if they existed with array hybridization, RNA degradation and data normalization. Microarray data were preprocessed using the mmgMOS normalization method [41, 42] using the default settings and differential expression (DE) was calculated using the *puma*DE method both implemented in the Bioconductor package “*puma*” [42–45]. The *puma* method uses a Bayesian hierarchical model to calculate the probability of positive likelihood ratio (PPLR). The PPLR associates probability values of genes being differentially expressed, which is a measure of false positive detection of DE, to each ratio and generates lists of genes ranked by the probability of DE. This PPLR statistic was converted into “*P-*like values” using the recommended formula in the puma method prior to subsequent analysis.

As many of the original annotations for the Affymetrix bovine chip are erroneous [6, 46], remapped annotations were determined using the “bovinedaiplusv6cdf” chip definition file (CDF). This annotation is based on the CDF-Merger procedure as described by De Leeuw et al. [47], which generates a hybrid CDF based on the standard Affymetrix CDF (version 26) and the custom Brainarray (version 11.0.1) CDF. This re-mapped annotation includes mapping to all RefSeq (mature RNA protein coding transcripts and validated complete coding sequences in GenBank). Annotations were also supplemented by interrogating the Ensembl *Bos taurus* database version 46 using the BioMart package in Bioconductor and manual annotation where possible with recent entries in Entrez Gene.

### Pathway analysis

To examine the molecular functions and genetic networks, the microarray data were further analyzed using Ingenuity Pathway Analysis (v. 8.8, Ingenuity Systems, Mountain View, CA; http://www.ingenuity.com), a web-based software application that enables identification of over-represented biological mechanisms, pathways, and functions most relevant to experimental datasets or genes of interest [40, 48–50].

A dataset containing gene identifiers and corresponding expression and *P*-like values was uploaded into IPA. Briefly, each identifier was mapped to its corresponding gene object in the Ingenuity knowledge base. A *P*-like value of *P* < 0.05 from the puma analysis was set to identify genes whose expression was statistically significantly up- or down-regulated. These genes, called “focus” genes, were overlaid onto a global molecular network developed from information contained within the Ingenuity knowledge base. Networks of these focus genes were then algorithmically generated based on their connectivity. Network analysis returns a score that ranks networks according to their degree of relevance to the network eligible molecules in the dataset. The score takes into account the number of network eligible molecules in the network and its size, as well as the total number of network eligible molecules analyzed and the total number of molecules in the knowledge base that could potentially be included in networks.

### RT-qPCR analysis

The microarray results were validated by carrying out RT-qPCR on 18 genes. Candidate genes were chosen based on the following criteria; those that were top ranking in our microarray DEG list, genes with known functional importance in uterine mediated sub-fertility which were either up- or down-regulated and genes which were not differentially expressed between the two treatment groups.

Using the same RNA samples that were analyzed in the microarray studies, first strand cDNA was synthesized using the High Capacity cDNA Reverse Transcription kit according to manufacturer’s instructions (Applied Biosciences, Ireland). Purified total RNA (1 μg) was reverse transcribed using random hexamers. The converted cDNA was quantified by absorbance at 260 nm, diluted to 50 ng/μl working stocks and stored at −20°C, for subsequent analyses.

Analysis of putative reference genes for RT-qPCR studies was carried out using GeNorm version 3.5 Microsoft Excel Add in (Microsoft, Redmond, WA) [51]. The stability of the expression of several cited reference genes including, ribosomal protein L15 [52], 18 s ribosomal RNA [53], ubiquitin [54], glyceraldehyde phosphate dehydrogenase and β-actin [55, 56], was investigated across all samples in this study. Similar to Coyne et al. [54], ubiquitin (at an optimal concentration of 2.5 μM) exhibited the greatest stability during qPCR analysis of endometrial mRNA samples analyzed, with an *M* value of 0.022. Based on a recommended cut-off *V* value of 0.15; ubiquitin was selected as a single standard reference gene for these experiments as the use of additional reference genes did not contribute to a more accurate normalization factor.

Primers were designed, to span exon boundaries where possible, using the Primer3 software programme [57] and oligos were aligned by Basic Local Alignment Search Tool (BLASTN) on the National Centre for Biotechnology Information (NCBI) web page, to verify their identity and homology to the bovine genome (http://www.ncbi.nlm.nih.gov/BLAST/). All oligonucleotides were commercially synthesized as highly purified salt-free products by Sigma Aldrich Ireland Ltd. Primers were first tested using end point PCR to optimize amplification conditions. All amplified PCR products generated in this study were purified using the PCR purification kit (Roche, Basel, Switzerland) and sequenced (Macrogen; Nucleics Pty Ltd, Bendigo, Australia) to verify their identity. Primer sequences used in this study are listed in Table 1.

**Table 1:** **Bovine specific oligonucleotide forward and reverse primer sequences (5′-3′) and PCR product length**

Gene name	Sequence	Accession number	Amplicon size (bp)
*18S/28S*	F: 5′- TGCTCTCGCAAACCTAACCT-3′	DQ222453	159
	R: 5′- CACTAAGCACTCGCATTCCA-3′		
*ACTA2*	F: 5′- ACTGGGACGACATGGAAAAG -3′	BT021508	166
	R: 5′- TACATGGCTGGGACATTGAA-3′		
*ACTB*	F: 5′- ACTTGCGCAGAAAACGAGAT-3′	BT030480	121
	R: 5′-CACCTTCACCGTTCCAGTTT-3′		
*APEH*	F: 5′- CAAGAGCATGCGCAGTATGT -3′	BC123400	181
	R: 5′- GTAGAGCTGCAAAGCCCATC-3′		
*CELA1*	F: 5′- GGAACCATCCTGGCTAACAA -3′	BC149525	165
	R: 5′- CATGGTGGTCTTCACAGTGG -3′		
*DONSON*	F: 5′- TGTGTTGGTGAAGGGAATGA -3′	BC133573.1	107
	R: 5′- AGAGGGTTGGTGGAAGTCCT -3′		
*FST*	F: 5′- TAAATGAGAGACCCGCCAAC-3′	AY775795	171
	R: 5′- CCCCAGTTTCTGTCCTGTGT-3′		
*GALNT6*	F: 5′- GACCACGTCTTGGACCTCAT-3′	NM_001015534	146
	R: 5′- AGCTCAGCTGGGGTGTAGAA-3′		
*GAPDH*	F: 5′- GGGTCATCATCTCTGCACCT-3′	NM_001034034	176
	R: 5′- GGTCATAAGTCCCTCCACGA-3′		
*GJA1*	F: 5′- - CAACATGGGTGACTGGAGTG 3′	BT021508	110
	R: 5′- GCAGGATTCGGAAAATGAAA -3′		
*IL33*	F: 5′- TTGTTTTGGAGGATGGAAGC -3′	BC123562	163
	R: 5′- TTTGTGGGGCTCAGGTTTAC -3′		
*MMP19*	F: 5′- TGGACGTTATCCCCTCAGTC-3′	BC123722	119
	R: 5′- GTCCATGGTTCATGCTTGTG -3′		
*MOSC2*	F: 5′- GCAGTGCTTTTGAGGAGGAC-3′	NM_001076380	169
	R: 5′- GGATCACACAGGCGGTAACT-3′		
*NMB*	F: 5′- ACATGACGACATGGCTGAAA-3′	NM_001075270	185
	R: 5′- ACTTCAACAGGGAAGCGAGA-3′		
*NPPC*	F: 5′- GAGGCAACAAGAAGGGTTTG -3′	BC123399	149
	R: 5′- CTGATGACCAAGGGTGACCT -3′		
*PPARA*	F: 5′- TTGTGGCTGCTATCATTTGC-3′	AF229356	135
	R: 5′- AGAGGAAGACGTCGTCAGGA-3′		
*RAB3B*	F: 5′- TGGGCGGAGATTCATTTTAC -3′	BC112795	144
	R: 5′- GAAAAGTGTGCATGGGTGTG -3′		
*RPL15*	F: 5′- TGCATAAGCACAGGGAGATG-3′	BT020706	134
	R: 5′-CTGGAGAGTATTGCGCCTTC-3′		
*SFRP1*	F: 5′- GTCCCTCTGGGTGAATCTGA-3′	NM_174460	158
	R: 5′- TCACTAATTGCCAGGGGTTC-3′		
*SLC1A3*	F: 5′-CATCCATGCTGTCATTGTCC-3′	BC120125	188
	R: 5′-ATCTGGTAACGCGTTTGTCC -3′		
*SLC45A2*	F: 5′- CATGCCCTCTTCACAGGTTT-3′	XM_001251343	179
	R: 5′- AGTGGGGCTTCAGGGATACT -3′		
*TGFB1I1*	F: 5′- CCTGCAATAAACCCATTGCT-3′	NM_001035313	162
	R: 5′- AGAAGCGCTCGAAGTAGCAC-3′		
*UBQ*	F: 5′- TACAACAGTTGGTGGCCAAA-3′	BC102888	121
	R: 5′-GAAGACTGGGCTGACTGAGG-3′		

18S/28S, 18S/28S ribosomal RNA; ACTA2, actin alpha 2; ACTB, beta-actin; APEH, N-acylaminoacyl-peptide Hydrolase; CELA1, chymotrypsin-like elastase family member 1; DONSON, downstream neighbor of SON; FST, follistatin; GALNT6, UDP-N-acetyl-alpha-D-galactosamine:polypeptide N-acetylgalactosaminyltransferase 6; GAPDH, glyceraldehyde-3-phosphate dehydrogenase; GJA1, gap junction protein, alpha 1; IL33, interleukin 33; MMP19, matrix metallopeptidase 19; MOSC2, MOCO sulphurase C-terminal domain containing 2; NMB, neuromedin B; NPPC, natriuretic peptide C; PPARA, peroxisome proliferator-activated receptor alpha; RAB3B, RAB3B member ras oncogene family; RPL15, ribosomal protein L15; SFRP1, secreted frizzled-related protein 1; SLC1A3, solute carrier family 1 member 3; SLC45A2, solute carrier family 45 member 2; TGFB1I1, transforming growth factor beta 1 induced transcript 1; UBQ, ubiquitin.

Primer concentrations were optimized for each gene by titrating 5, 10, and 20 μM per primer. The most suitable primer concentration was chosen based on four criteria in order of decreasing importance: i) a clear distinct melt curve absent of any additional peak(s) caused by non-specific binding, ii) a curve within the temperature range 75–85°C, iii) the primer concentration producing the lowest threshold cycle number (*C*_t_) and lastly, iv) replication amongst *C*_t_ values and melting temperatures (T_m_). Subsequently, efficiencies of chosen primer concentrations were determined over a 5-fold dilution series, whereby cDNA was diluted into working solutions: stock, 1:2, 1:4, 1:8, 1:16, and RT-qPCR assays carried out. This was repeated for every gene. The *r*^2^ and amplification efficiency (*E*) values for RT-qPCR were calculated from linear regression analysis of log (input cDNA) versus *C*_t_ plot. The slope for each set of standards was used to determine *E* = 10^(−1/slope)^ – 1. Slopes, amplification efficiencies and R^2^ estimates for individual genes are reported in Table 2. Only primers with PCR efficiencies between 90% and 110% were used.

**Table 2:** **Efficiency variables for individual RT-qPCR genes**

Gene	Optimum [primer] μM	Slope (−)	R ^2^	Efficiency
*ACTA2*	5	3.62	0.99	1.890
*APEH*	20	3.18	0.99	2.064
*CELA1*	10	3.54	0.99	1.917
*DONSON*	10	3.20	0.99	2.054
*FST*	2.5	3.60	0.97	1.896
*GALNT6*	5	3.29	0.99	2.103
*GJA1*	20	3.31	0.98	2.003
*IL33*	5	3.33	0.96	1.997
*MMP19*	10	3.52	0.99	1.925
*MOSC2*	10	3.14	0.99	2.082
*NMB*	10	3.21	0.99	2.049
*NPPC*	5	3.63	0.99	1.885
*PPARA*	5	3.33	0.99	1.997
*RAB3B*	20	3.51	0.99	1.927
*SFRP1*	5	3.70	0.97	1.863
*SLC1A3*	10	3.46	0.97	1.946
*SLC45A2*	10	3.54	0.95	1.916
*TGFB1I1*	20	3.29	0.96	2.013
*UBQ*	2.5	3.20	0.99	2.054

Each RT-qPCR reaction was carried out in a 96-well plate format with a total volume of 20 μl, containing 1 μl cDNA, (10 ng/μl), 10 μl Fast SYBR® Green Master Mix (Applied Biosystems, Ireland), 1 μl forward and reverse primers and 8 μl nuclease-free H_2_O. Performance of RT-qPCR was carried out using the Applied Biosystems Fast 7500 v2.0.1 with the following cycling parameters: 95°C for 10 min followed by 40 cycles of 95°C for 15 s and 60°C for 60 s, followed by amplicon dissociation (95°C for 15 s, 60°C for 60 s, 95°C for 15 s and 60°C for 15 s). Dissociation curves were examined for the presence of a single PCR product. The software package GenEx 5.2.1.3 (MultiD Analyses AB, Gothenburg, Sweden) was used for efficiency correction of the raw cycle threshold (Ct) values, interplate calibration based on a calibrator sample included on all plates, averaging of replicates, normalization to the reference gene and the calculation of quantities relative to the greatest Ct. Expression of each target gene was normalised to the reference gene and relative differences in gene expression were calculated using the 2^-ΔΔCT^ method [58].

### Statistical analysis

All data were analyzed using the Statistical Analysis Systems software package (SAS Inst. Inc., Cary, NC) version 9.1. Data from RT-qPCR studies were tested for adherence to normality using PROC UNIVARIATE (SAS, 2003). Non-normal data were subsequently transformed using the best fit function as described by PROC TRANSREG (SAS, 2003). Differences in mean values between the two groups (HF and LF) were tested using ANOVA (PROC MIXED). Animal within treatment was used as the error term. The Tukey critical difference test was used to determine statistical difference between LF and HF mean values. The CORR procedure of SAS (PROC CORR, SAS 2003) was used to determine correlations between microarray and RT-qPCR data. Pearson correlation coefficients were estimated for each individual gene across all animals (n = 12). A *P* value of *P <* 0.05 was considered to be statistically significant. Data collected from CL diameter measurements were tested for adherence to normality using PROC UNIVARIATE (SAS, 2003). CL differences in mean values between the two groups (HF and LF) were tested using ANOVA (PROC MIXED). Animal within treatment was used as the error term. For the analysis of progesterone profiles individual profiles were normalized relative to day of estrus (Day 0). The effect of fertility status “HF” versus “LF” was established using a repeated measured analysis (PROC MIXED; SAS).

## Results

### Animal model

Embryo survival rates were 73.3%, 71.7%, 73.3% and 70.0% for A.I. rounds 1, 2, 3 and 4 respectively. A total of 31 heifers qualified as HF or LF; 15 HF and 16 LF, of which three of these were eliminated from the study due to the presence of ovarian abnormalities detected at ultrasound scanning. Pregnancy rate for LF heifers was consistent across all four replicates. Six HF and 6 LF heifers were randomly chosen within their respective fertility groups for slaughter on D7. The mean inter-estrous intervals in a previous recorded estrous cycle were 20.17 ± 0.96 and 20.83 ± 0.96 days (*P* > 0.10) for the HF and LF heifers, respectively. At day of slaughter, mean CL diameters were 22.58 ± 3.48 (SD) mm and 23.55 ± 4.4 (SD) mm for HF and LF heifers, respectively, i.e., there was no significant difference in CL diameter between fertility groups (*P* > 0.10).

### Progesterone profiles

There was no effect of fertility status, or interaction effect of fertility status and day of cycle (*P* > 0.10), on the concentration of progesterone. On the day of slaughter plasma concentrations did not differ between the high and low fertility groups (HF 5.96 ng ml^−1^; LF 5.65 ng ml^−1^, *P* = 0.589).

### Microarray differential gene expression

A total of 419 genes were found to be differentially expressed between LF and HF (n = 6 vs. 6). Of these, 171 were up-regulated and 248 down-regulated in the LF compared with HF heifers, respectively. Transcript abundance differences between LF and HF groups resulted in fold changes ranging from 6.6-fold down to 8-fold up-regulated in LF animals. The microarray data have been deposited in NCBI’s Gene Expression Omnibus [59] and are accessible through GEO Series accession number GSE29853. Hierarchical clustering of differentailly expressed genes is presented as a heatmap and dendogram in Additional file 1: Figure S1.

### Pathway analysis

Of the 419 DEG, a total of 227 genes were successfully mapped to a molecular/biological pathway and/or category in the IPA database, while 202 of these were network eligible using IPA. Among the mapped DEG, 73 were up-regulated (Additional file 2: Table S1) and 154 down regulated (Additional file 2: Table S2).

### Biological functions

**Figure 2:**
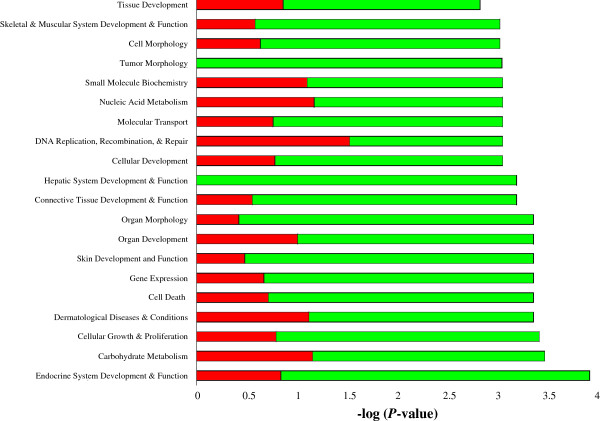
**Classification of DEG according to top 20 molecular and cellular functions, most significantly affected by endometrial related sub-fertility, using IPA.** The red/green bars indicate the likelihood [−log (P-value)] that the specific molecular and cellular function category was affected by endometrial related sub-fertility compared with others represented in the list of DEG. The proportion of up- and down-regulated genes in each group is represented by the red and green segments on each bar, respectively.

Biological categories with the largest number of up regulated genes included DNA replication, recombination and repair, nucleic acid metabolism and carbohydrate metabolism. Categories with the largest number of down-regulated genes were organ morphology, and connective tissue development and function. Of the top 20 most statistically significantly over-represented biological categories, DNA replication, recombination and repair had the greatest ratio of up- to down-regulated genes (Figure 2). Pathways with the greatest number of DEG, including their respective number of DEG, were cellular growth and proliferation (n = 57), inflammatory disease (n = 55), cell death (n = 49), cellular development (n = 43), small molecule biochemistry (n = 37), cellular morphology (n = 36) and tissue development (n = 36) as shown in Table 3.

**Table 3:** **Biological categories from IPA analysis with the largest number of DEG**

Biological category	Gene symbol
Cellular growth and proliferation	*ACTB, ADCY3, CAT, CD58, CIAO1, CNOT8, COL1A2, CTSL2, CXCL2, CTSL2, DAP, DCK, EMP3, ENPP1, ETFDH, FASN, FHL2, FST, FUS, GADD45B, GJA1, GLI3, HMGB1, IL6ST, ILF3, IMPDH2, LGALS1, LGALS3, LTBP1, LY6E, MAGED1, MAP3K7IP2, MEST, MMP19, NMB, ODC1, PARK7, PPARA, PHLDA1, PPARA, PRKRA, PRMT1, PSMB10, PSMD2, PTPRK, RBBP7, SERPINH1, SFRP1, SLC1A3, SOX6, TCF12, TGFB1I1, TOB2, TP53I11, UCP3, WFDC1, ZNF259*
Inflammatory disease	*ACTA2, ACTB, ADCY2, ADCY3, APEH, C1QTNF6, CACNB2, CAT, CD58, CORO2A, CXCL2, DAP, DCK, DSC2, EEF2, ENPP1, FAU, FNBP1, GADD45B, GALNT2, GLI3, GLUL, GSDMB, HMGB1, IFI6, IL33, IL6ST, IMPDH2, LGALS1, LGALS3, LPHN2, LTBP1, MYLK, NSF, ODC1, ORC5L, PARP4, PCMTD1, PPARA, PRMT1, PTGIS, PTPRK, RAB12, RARRES2, RGNEF, RPS3, SCG5, SLC1A3, SLC25A24, SLC45A2, SOX6, SRPK2, TCF12, TNIK, WFDC1*
Cell death	*ACTB, ACTC1, BAG3, CACNB2, CAT, CTSL2, CXCL2, DAP, EMP3, FASN, FAU, FHL2, FST, FUS, GADD45B, GALNT2, GIMAP5, GJA1, GLI3, HMGB1, HSPB1, IFI6, IL6ST, LGALS1, LGALS3, LTBP1, MAGED1, MAP3K7IP2, MYLK, NPPC, NSF, ODC1, PARK7, PARP4, PHLDA1, PPARA, PRKRA, PTGIS, QKI, RCAN2, RPS3, SCG5, SFRP1, SLC1A3, SLC25A24, SOX6, SRPK2, TCF12, TGFB1I1*
Cellular development	*ARHGDIG, ARHGEF11, BOC, CAT, CTSL2, CXCL2, ENPP1, FHL2, FHL3, FLNC, FST, FUS, GADD45B, GEFT, GIMAP5, GJA1, GLI3, HMGB1, IL33, IL6ST, LGALS1, LGALS3, LTBP1, MAGED1, MARCKS, MYLK, NPPC, NR0B1, ODC1, PPARA, QKI, RARRES2, RCAN2, RNF128, SFRP1, SLC1A3, SMOC2, SOX6, STIM1, TCF12, TGFB1I1, TNIK, TOB2*
Small molecule biochemistry	*ACAT1, ANKRD26, CAT, CMPK1, DCK, ERH, ETFDH, FASN, FST, GALNT2, GJA1, GLUL, HMGB1, IL6ST, LGALS1, LRAT, MARCKS, NMB, NPPC, ODC1, PAICS, PARK7, PCCB, PPARA, PRMT1, PTGIS, QKI, RAB3B, SCG5, SLC1A3, SLC25A12, SOX6, SRD5A1, SULT1A1, SV2A, TGFB1I1, UCP3*
Cell morphology	*ACTA2, ACTC1, ANXA6, ARHGDIG, ARHGEF11, CTSL2, CXCL2, DPYSL3, ENPP1, FASN, FERMT2, FHL3, FLNC, FST, GJA1, HMGB1, HSPB1, IL6ST, LGALS1, LGALS3, LRAT, MARCKS, NPPC, NTN4, ODC1, PHLDA1, PPARA, RGNEF, RNF128, SCG5, SERPINH1, SFRP1, SLC1A3, SOX6, TGFB1I1, TNIK*
Tissue development	*ACTA2, ACTC1, ALX1, ARHGDIG, BOC, CAT, CD58, COL16A1, CXCL2, DSC2, ENPP1, FASN, FHL3, GADD45B, GEFT, GJA1, GLI3, HMGB1, IL33, IL6ST, LGALS3, LY6E, MARCKS, MEST, MMP19, NPPC, NR0B1, NTN4, PHLDA1, PPARA, PTPRK, SEMA5A, SFRP1, SOX6, TCF12, TGFB1I1*

### Canonical pathways

Canonical signaling pathway analysis uncovered genes with functions in ILK-signaling, TR/RXR activation, regulation of actin based motility by Rho and Integrin signaling (Table 4). Genes associated with canonical signaling pathways were down-regulated in LF animals for all statistically significant pathways mapped with the exception of TR/RXR activation where the ratio of up- to down-regulated genes was uniform. Canonical metabolic pathways over-represented within the microarray data included fatty acid biosynthesis, o-glycan biosynthesis and purine metabolism. There were more genes up-regulated in canonical metabolic than canonical signaling pathways with the greatest ratio of up- to down-regulated genes expressed in the metabolic pathway: o-glycan biosynthesis (Table 4).

**Table 4:** **Enriched canonical pathways in endometrial mRNA from HF and LF heifers**

Pathways	Genes	% DEG	***P*** -value
***Canonical signalling***			
Regulation of Actin-based Motility by Rho	*MYLK, ACTB, ACTA2, RHOU, ACTC1, FNBP*	6.5	0.0007
ILK Signalling	*TGFB1I1, FLNC, ACTN2, ACTB, ACTA2, FERMT2, RHOU, ACTC1, FNBP1*	4.3	0.0017
Caveolar-mediated Endocytosis Signalling	*FLNC, ACTB, ACTA2, ACTC1, COPB2*	6.0	0.0024
Polyamine Regulation in Colon Cancer	*PSMD11, PSMB10, PSMD2, ODC1*	7.0	0.0041
Cellular Effects of Sildenafil	*MYLK, ADCY2, ACTB, ACTA2, ADCY3, ACTC1*	3.9	0.0068
RhoA Signalling	*MYLK, ACTB, ACTA2, ARHGEF11, ACTC1*	4.5	0.0126
Mechanisms of Viral Exit from Host Cells	*ACTB, ACTA2, ACTC1*	6.8	0.0135
TR/RXR Activation	***UCP3*** *,* ***RAB3B*** *, FASN, RCAN2*	4.1	0.0245
Virus Entry via Endocytic Pathways	*FLNC, ACTB, ACTA2, ACTC1*	4.2	0.0245
Semaphorin Signalling in Neurons	*DPYSL3, RHOU, FNBP1*	5.8	0.0288
HMGB1 Signalling	*HMGB1 (includes EG:3146), RBBP7, RHOU, FNBP1*	4.1	0.0302
Thrombin Signalling	*MYLK, ADCY2, ADCY3, RHOU, ARHGEF11, FNBP1*	2.9	0.0331
Integrin Signalling	*MYLK, ACTB, ACTA2, RHOU, ACTC1, FNBP1*	3.0	0.0331
Sphingosine-1-phosphate Signalling	*ADCY2, ADCY3, RHOU, FNBP1*	3.6	0.0407
Germ Cell-Sertoli Cell Junction Signalling	*ACTB, ACTA2, RHOU, ACTC1, FNBP1*	3.2	0.0417
CXCR4 Signalling	*ADCY2, ADCY3, RHOU, ARHGEF11, FNBP1*	3.0	0.0447
***Canonical metabolic***			
Purine Metabolism	***NSF*** *, ADCY2, ENPP1, IMPDH2,* ***DCK*** *, ATP13A5, ADCY3,* ***PDE6C*** *, PAICS, POLR2H, ACTC1*	2.5	0.0019
Glycan Biosynthesis	*GALNT2,* ***GYLTL1B*** *,* ***GALNT6***	6.3	0.0077
Fatty Acid Biosynthesis	*FASN,* ***PCCB***	3.9	0.0098
Sulphur Metabolism	*SULT1A1, SUOX*	3.3	0.0316
Pantothenate and CoA Biosynthesis	*ENPP1, DPYSL3*	3.1	0.0347
Pyrimidine Metabolism	*ENPP1,* ***DCK*** *, DPYSL3, POLR2H, CMPK1*	2.2	0.0457

Genes marked in bold are up-regulated.

### Networks

**Figure 3:**
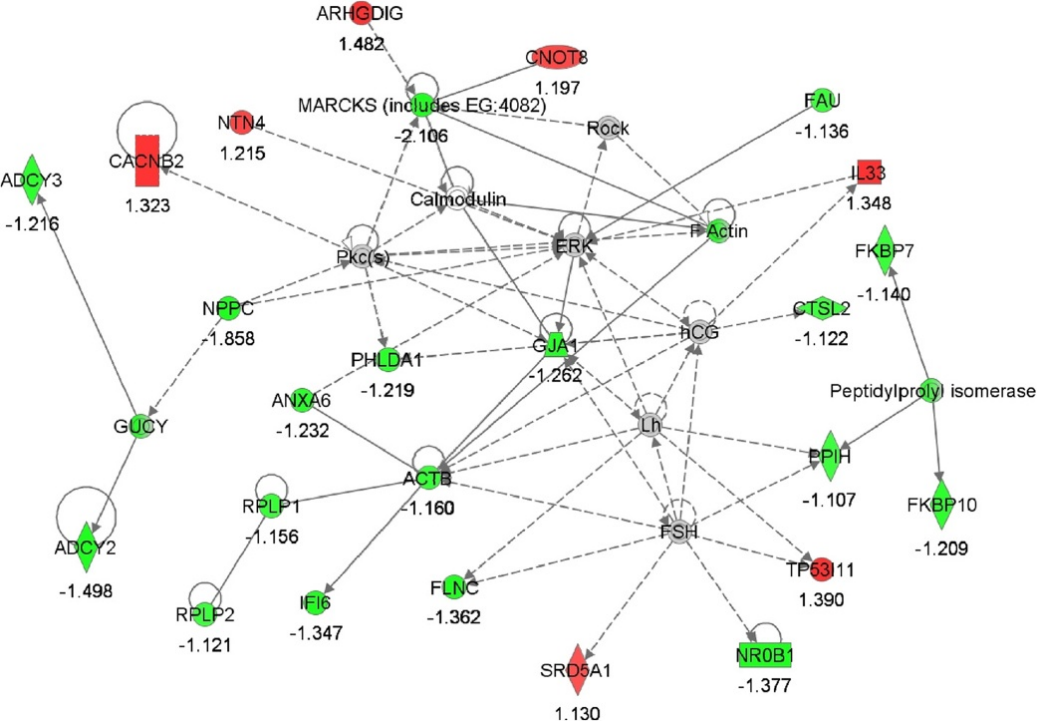
**Network #1; lipid metabolism, small molecule biochemistry.** The network is displayed graphically as nodes (genes). The node color intensity indicates the expression of genes; with red representing up-regulation and green, down-regulation in LF versus HF endometrium. The fold value is indicated under each node.

**Figure 4:**
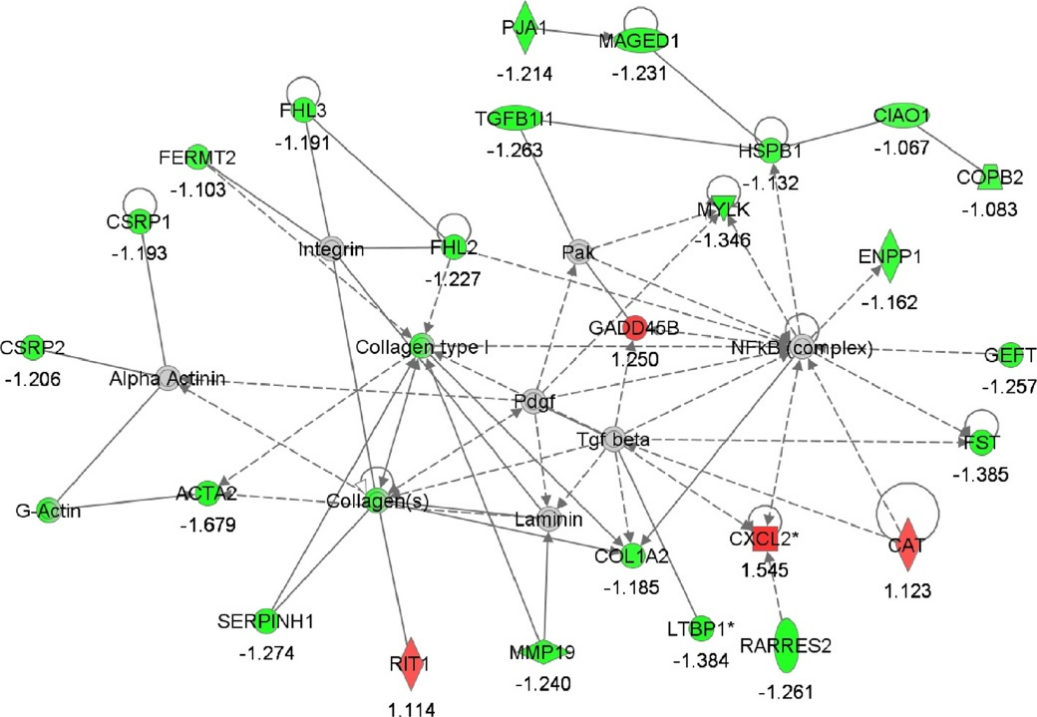
**Network #2; cellular growth and proliferation, connective tissue development and function, skeletal and muscular system development and function.** The network is displayed graphically as nodes (genes). The node color intensity indicates the expression of genes; with red representing up-regulation and green, down-regulation in LF versus HF endometrium. The fold value is indicated under each node.

Using IPA a total of 19 gene networks were identified, 12 of which had 13 to 25 focus genes among DEG (Additional file 2: Tables S1 and S2). The 12 top networks are listed in Table 5. Lipid metabolism featured in three of the top 12 networks. In addition, organ/tissue/cell morphology and development appeared a central biological theme over-represented among DEG. Illustrations of gene interactions among DEG contained within the top two scoring networks can be seen in Figures 3 and 4. Biological pathways; lipid metabolism, cell growth and proliferation, and tissue development and function, were repeatedly featured pathways that constituted these top scoring networks.

**Table 5:** **Networks generated from endometrial gene expression data of HF versus LF heifers by IPA**

Network ID	Top functions	Molecules in network	Score	Focus molecules
1	Lipid Metabolism, Small Molecule Biochemistry	*ACTB, ADCY2, ADCY3, ANXA6, ARHGDIG, CACNB2, Calmodulin, CNOT8, CTSL2, ERK, F Actin, FAU, FKBP7, FKBP10, FLNC, FSH, GJA1, GUCY, hCG, IFI6, IL33, Lh, MARCKS (includes EG:4082), NPPC, NR0B1, NTN4, Peptidylprolyl isomerase, PHLDA1, Pkc(s), PPIH, Rock, RPLP1, RPLP2, SRD5A1, TP53I11*	45	25
2	Cellular Growth and Proliferation, Connective Tissue Development and Function, Skeletal and Muscular System Development and Function	*ACTA2, Alpha Actinin, CAT, CIAO1, COL1A2, Collagen type I, Collagen(s), COPB2, CSRP1, CSRP2, CXCL2, ENPP1, FERMT2, FHL2, FHL3, FST, G-Actin, GADD45B, GEFT, HSPB1, Integrin, Laminin, LTBP1, MAGED1, MMP19, MYLK, NFkB (complex), Pak, Pdgf, PJA1, RARRES2, RIT1, SERPINH1, Tgf beta, TGFB1I1*	45	25
3	Carbohydrate Metabolism, Haematological Disease, Metabolic Disease	*26 s Proteasome, Akt, AMPK, EIF2C4, ELOVL5, FASN, GLUL, HISTONE, Histone h3, Histone h4, HMG20B, Hsp90, MAP3K7IP2, MED13, MED27, N-cor, ODC1, PPARA, PSMB10, PSMD2, PSMD11, PTPRK, RAB3B, RBBP7, RCAN2, RPL23, RPS3, RPS5, SFRP1, SMOC2, SOX6, SRPK2, T3-TR-RXR, Ubiquitin, UCP3*	44	25
4	Cell Death, Gene Expression, Lipid Metabolism	*ACAT1, Caspase, CD58, CMPK1, ERK1/2, FUS, HMGB1 (includes EG:3146), IFN Beta, IgG, IL1, IL12 (complex), IL6ST, ILF3, IMPDH2, Insulin, Interferon alpha, Jnk, LDL, LGALS1, LGALS3, LRAT, Mapk, Mek, NES, P38 MAPK, PCCB, PDGF BB, PI3K, PIBF1, PRMT1, RABEP1, Ras, SLC25A12, STAT5a/b, TNIK*	28	18
5	Cell Morphology, Inflammatory Response, Lipid Metabolism	*ARHGDIG, ARHGEF, ARHGEF11, CPS1, EMP3, ETFDH, FAU, FNBP1, GLI3, IFI6, LARP1, LEP, Lpa receptor, P2RX7, Pka, PLXNB2, PXN, RAGE, Ras homolog, RCN3, RGNEF, RHOH, RHOU, RHPN1, RND2, RPL8, RPL26, RPL29 (includes EG:6159), RPLP2, SCAMP2, St3gal, ST3GAL3, TNF, UCP3, YWHAZ*	27	17
6	Drug Metabolism, Small Molecule Biochemistry, Cell-To-Cell Signalling and Interaction	*ABHD5, ALDH3A2, BAG3, BCL2, beta-estradiol, BIK, BOC, C1QTNF6, CDON, CTNNB1, CTSH, CXADR, EEF2, F12, FOLH1, GADD45B, GIMAP5, HOXC6, HSPB8, IFT122, IGFBP6, KCNMB1, METTL7A, MME, MRVI1, PLIN2, PLIN5, PNPLA2, PTGIS, PTPRK, PTPRU, SEMA5A, SFRP1, SULT1A1, SULT1A3*	25	17
7	Embryonic Development, Organ Development, Organ Morphology	*Arginase, AZGP1, BMP6, C12ORF11, C19ORF10, C21ORF7, CALM2, COL16A1, COL4A6, CSRP2, CTSH, CTSL2, DBNDD2, DPYSL3, DYRK2, ENPP1, GOLGA7, HRAS, HTT, IFNG, IL13, LPHN2, LRBA, LY6E, Pdgf, PLOD1, RAB12, RAB33A, SLC1A3, SRM, SV2A, TCF12, TGFB1, UNC5B, ZDHHC9*	23	15
8	Developmental Disorder, Neurological Disease, Cell Death	*ADAM10, AHCYL2, BCL2L14, CABC1, CORO2A, DUT (includes EG:1854), ERH, F11R, GRPR, LETMD1, MAPK1, MARCKS (includes EG:4082), MEST, NFATC2IP, NMB, NMBR, NMT1, NR3C1, PARK7, PLK3, PMM1, PRKRA, PRPSAP1, REEP5, RNF144B, SH3D19, SLC45A2, SMN1, SNUPN, SPOP, TP53, TRAF6, UBE2T, ZNF259, ZNF346*	22	15
9	Cellular Development, Cell Cycle, Connective Tissue Development and Function	*ABCD3, ACTC1, Actin, ATP13A5, ATP5J2, ATPase, CDKN2A, CLPX, DDX19B, DONSON, FAM167A, GPHN, HIP1R, HLTF, KATNA1, MED27, MIR373, MIR297-2, NCALD, NSF, ORC5L, PACRGL, PHACTR1, phosphatidylinositol 3,4-diphosphate, PLS1, POLR2H, RNA polymerase II, RSPO1, SDCCAG3 (includes EG:10807), SNX16, SUOX, TBX3, TERT, TOB2, VIM*	21	14
10	Post-Translational Modification, Cell Cycle, Gene Expression	*ACAA1B, CPT2, CUEDC2, DAP, EHHADH, ERBB2, ESR1, FRRS1, GALNT1, GALNT2, GALNT3, GALNT6, HCG 2023776, IGFBP6, LAMP1, LAMP2, MMS19, MRC1, MYC, NCOA4, OLFML3, PAICS, PHF5A, Polypeptide N-acetylgalactosaminyltransferase, PPARG, PTRF, QKI, RAB34, SCG5, SETDB1, SFRP1, STIM1, TPD52, WWC1*	19	13
11	Cancer, Cardiovascular Disease, Cell-To-Cell Signalling and Interaction	*ALX1, APEH, C20ORF160, COPB2, COX2, COX4I1, DAG1, EPO, FOS, GMFB, GRB2, GRP, GSTK1, GYLTL1B, HCLS1, HNRNPH2, HNRNPR, Hydrolase, KHSRP, MST1R, ONECUT1, P2RY1, PDE6C, PDE6G, POP7, RAB3GAP1, RCC2, RNF128, RPL7, RPS7, RPS18, SMARCD2, TMEM62, USP8, YPEL5*	19	13
12	Embryonic Development, Tissue Development, Tissue Morphology	*ACOT13, APBB1, BACE1, C11ORF52, CBS, CELA1, Coup-Tf, DCK, DKK1, DSC2, FOXA1, HMGB2, HNF4A, IER5L, JKAMP, KRR1, LRP, LRP2, LRP5, LRP6, MESDC2, NR2F1, NR2F2, PARP4, PHB (includes EG:5245), PKP2, Plasminogen Activator, POU5F1, RSPRY1, Secretase gamma, SLC17A5, TXNDC12, UBE2D3 (includes EG:7323), UBE2V1, WNT4*	19	13

### RT-qPCR analysis

**Figure 5:**
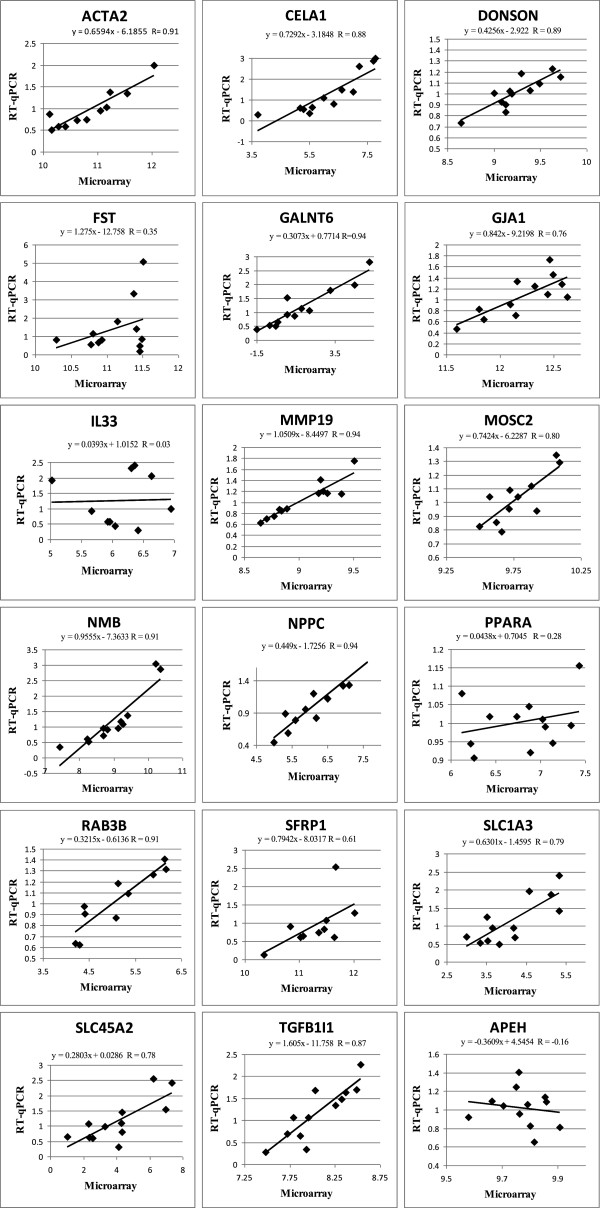
**Genes validated between RT-qPCR and microarray methodologies, including correlation coefficients (R) (n=12).**

Eighteen genes were validated by real-time RT-qPCR (Table 1). There was moderate to good consistency between methodologies for direction and magnitude of differential gene expression among genes analyzed. Correlation coefficients exceeded 0.60 in fourteen of the eighteen genes validated (Figure 5, Additional file 2: Table S3).

## Discussion

The animal model generated in this study, is the first of its kind. Two groups of heifers consistently divergent in conception rate; HF and LF were successfully generated and endometrial gene expression examined. We identified key genes and pathways potentially contributing to endometrial related conception rate variance, the most extreme of which had no previously known involvement in endometrial function, including cellular growth and proliferation *NPPC* and *GJA1;* angiogenesis *MMP19* and *HMGB1*; lipid metabolism *FASN* and *PPARA*; cellular and tissue morphology and development *FST* and *TGFB1I1*; inflammation *IL-33*; and metabolic exchange *SLC1A3* and *SLC25A24*.

Several studies have highlighted the vital role progesterone plays in early embryo development to the extent that decreased conception rates were observed in heifers with a delayed postovulatory progesterone peak [60]. Furthermore, it has been well documented that progesterone influences endometrial and oviductal function [61, 62]. In the present study, progesterone concentrations were within the normal range for both HF and LF heifers, and did not vary between groups. In addition, CL diameter measurements were not different between HF and LF animals and were consistent with observations from other studies examining CL diameters during this period of the estrous cycle [63]. The high conception rates achieved across successive breedings was indicative of reproductively healthy animals, with good heat detection and insemination technique providing confidence in retrospective fertility status. However, it is important to note, other factors potentially contributing to the conception rate differences observed between HF and LF heifers, including oocyte quality and oviductal environment, were not analysed in this study.

Endometrial function plays a critical role in pre-implantation embryo survival. Consequently, much work has focused on the biochemical and molecular phenomena surrounding the progression of an estrous cycle [27]. The present study is novel as it provides information on gene expression during an important period of the estrous cycle: the mid-luteal phase, otherwise recognized as a critical period of embryo loss during pregnancy [5, 7] between animals of high and low reproductive capacity. Reiterating the importance of examining transcription during this phase, Salilew-Wondim et al. [31] recently found more extensive differential gene expression in endometrium harvested from heifers on D7 (an estrous cycle prior to embryo transfer) between heifers that conceived and those that returned to estrus before day 21, when compared with D14.

*GALNT6*, encoding enzyme UDP-N-acetyl-alpha-D-galactosamine: polypeptide N-acetylgalactosaminyltransferase 6, was the most abundantly expressed gene in LF heifers. It was 6.7 fold up-regulated in LF compared with HF heifers. This is the first report of expression of this gene in *Bos taurus*. The *GALNT6* gene is located on chromosome 5 in the bovine genome and shares a coding region with SLC4A8, a sodium bicarbonate co-transporter. Expression of this gene in humans is implicated in the synthesis of oncofetal fibronectin (onfFN) [64], a protein found in plasma and cervicovaginal secretions; increased concentrations of which has been associated with abnormal pregnancy [65, 66]. However, Feinberg et al. [67] reported increased protein levels of onfFN at the trophoblast–endometrial ECM interface in human pregnancy tissues from gestational day 20 to full term in healthy pregnancies. These observations suggest that differential expression of the enzyme *GALNT6* may have consequences for embryo survival and that this may be time specific however its role is currently unclear.

Pathway analysis is widely used to analyze gene expression data and serves as an effective tool for delineating the underlying biological processes involved in mRNA aberrations [68–71]. Biological pathways altered in the current study included: cellular growth and proliferation, lipid metabolism, tissue remodeling, ECM mineralization, inflammation, angiogenesis, and metabolic exchange.

### Cellular growth and proliferation

Owing to its regenerative nature, the endometrium undergoes highly complex but tightly regulated cellular proliferation and differentiation throughout the estrous cycle [72]. There is little published information on the molecular mechanism of bovine endometrial proliferation throughout the estrous cycle however, studies examining uterine tissue of non-pregnant ewes during cycle days 0 to 15 showed an increased rate of cellular proliferation between days 0 and 4, decreasing by day 15, suggesting a proliferative disposition is normal earlier in the estrous cycle [73]. Results from our study indicate that LF animals could be experiencing an abnormal decline in cellular growth/proliferation i.e. 21 genes implicated in cellular proliferation inhibition, including *FST*[74], *NPPC*[75], *GJA1*[76], *SOX6*[77], were up-regulated in the LF animals. Of these genes *FST*, *NPPC* and *GJA1* were previously found to be expressed in bovine endometrial tissue [78, 79]. Substantial inhibition of endometrial cellular proliferation would retard the development of a secretory endometrium and suppress endometrial maturation [80], thus making successful implantation unlikely.

### Angiogenesis

A critical element of tissue growth and development is the growth of new blood vessels, also known as angiogenesis [81]. Generally inactive in healthy individuals and animals, angiogenesis plays an active role in endometrial function, as well as growth of ovarian follicles and CL during the reproductive cycle [82, 83]. In a highly proliferating tissue such as endometrium, and particularly during the hypothesized window of proliferation day 0 to 14/15, angiogenesis is necessary for the provision of nutrients. Factors controlling angiogenesis include growth factors, nitric oxide and matrix metalloproteinases (MMPs), of which *MMP19* was down-regulated in the LF animals [84]. Also down-regulated, high-mobility group box 1 (*HMGB1*) which codes for a protein which has previously been identified in uterine fluid of dairy heifers on day 7 post estrus [22]. A role for members of the HMBG family in angiogenesis is supported by their expression during mouse embryogenesis [85] with higher expression levels found in proliferating cells [86] and lower expression in fibroblasts from old-age humans [87]. Down-regulation of these and other angiogenic genes, which was the case in LF animals, could prevent the necessary angiogenic cascades synergistic with cellular proliferation that dominate the mid-luteal phase [34].

### Lipid metabolism

Lipid metabolism appears in three of the top 5 networks, suggesting its importance as a metabolic process in uterine physiology. Genes involved included *ACAT1*, *CCAT*, *LGALS1*, *PCCB*, *SRD5A1*, *FASN* and *PPARA*. In particular, increased *PPARA* transcript abundance, as observed in LF heifers, coincides with increased fatty acid catabolism [88]. Fatty acids are essential precursors to steroids and eicosanoids, metabolites necessary for normal ovarian and uterine function [9]. Furthermore, studies have shown fatty acid supplementation positively influences reproductive performance [9, 54].

Fatty acid synthase (*FASN*) exhibits its anabolic capacity by aiding in the conversion of dietary carbohydrate to fat, which is subsequently organized into hepatic adipocytes and lactating mammary tissue as triglyceride and milk lipids, respectively [89, 90]. It has also been found that expression of *FASN* peaks during the proliferative phases of the menstrual cycle [91]. Metabolic demands are particularly high during this phase as a result of the extensive endometrial remodeling and reconstruction, a central theme to both the estrous and menstrual cycles. Increased *FASN* would be favorable in such a demanding situation to deliver the required fatty acid for the assembly of new cell membranes, modification of DNA transcriptional machinery and hormone construction. Interestingly, expression of *FASN* was down-regulated in the LF heifers suggesting the aforementioned processes were compromised in these animals, which potentially affecting their ability to conceive.

Steroid 5α-reductase type 1 enzyme is involved in the metabolism of progesterone that is found in uterine and cervical cavities. Murine gene knock-out studies have shown that parturition is adversely affected by aberrant expression of this gene, impeding cervical ripening and fetal delivery as a result of elevated progesterone levels in the cervix [92]. Expression of the gene coding for this enzyme was up-regulated in LF heifers, thus progesterone catabolism is likely to be active in these animals. As high progesterone levels are positively associated with embryo survival [60, 93], it is therefore possible that the LF animals are experiencing low local progesterone concentrations and ultimately, this could be contributing to their low conception rates.

### Cellular and tissue morphology and development

The ability of cells to generate alternate cell types whose phenotype is different from that of the source tissue is known as plasticity. Endometrial epithelial and stromal cell proliferation, as discussed previously, is a complex multi-component process involving cues from extra-cellular growth factors and ovarian hormones [72, 94]. However, in their absence, isolated bovine endometrial stromal cells exhibit the ability to develop into bone [95]. Results from our microarray study showed a large representation from this biological category, with 36 DEG enriched. Genes implicated in cell and tissue morphology and development which were down-regulated in low fertility heifers included, *PPARA*, *IL6ST*, *GJA1*, *SFRP1* and *IL-33*.

One particular biochemical pathway which facilitates cellular transformation includes extracellular matrix mineralization (ECM) [96]. A well known regulator of ECM mineralization is the activin a-FST system. Activin A inhibits ECM mineralization whereas *FST*, an activin antagonist which prevents activin-receptor interaction [97], increases mineralization in cell cultures [98]. Transgenic female mice with gain-of-function *FST*, in which mouse follistatin was over-expressed, developed thin uteri and small ovaries, resulting in infertility [99]. *FST* was differentially expressed between HF and LF heifers, indicating a role for this gene pathway in mid-luteal endometrial homeostasis and early embryo survival.

ECM remodeling, occurring during both pregnancy and the estrous cycle, facilitated by the matrix-metalloproteinases, ensures the provision of a suitable structural microenvironment where the embryo can grow [100, 101]. Matrix-metalloproteinase-19 (*MMP-19*), an important molecule in this pathway and which was down-regulated in LF heifers, plays a significant role in ECM remodelling [102]. Interestingly, Wathes et al. [103] reported that differential expression of genes *MMP* - *1*, *2*, *3*, and *13* two week post partum in the bovine endometrium, was highly correlated with differential expression of IGF binding protein 4, a known antagonist of *IGF1* expression [104]. The IGF system, in particular IGF1, is associated with several reproductive processes in cattle including preimplantation embryo development [105–107].

The transforming growth factor βs (TGF-β) are multifunctional cytokines that also regulate tissue remodelling and repair [108, 109]. High expression of TGF-β has been observed during pro-estrus and diestrus [110, 111] thereby highlighting the role for TGF-βs in endometrial remodelling, an important process impeding estrous cycle transition [112]. Transforming growth factor beta 1 induced transcript (*TGFB1I1*) was down-regulated in the LF animals, suggesting altered or irregular endometrial remodelling in these animals which may be contributing to the conception rate differences observed between the two divergent fertility groups.

### Inflammation

Inflammation is an innate cyclical physiological process facilitating progression of reproductive cycles in the endometrium. The animal model in this study isparticularly useful for the identification of inflammatory pathways associated with uterine low fertility for numerous reasons. Firstly, there has been no mitogenic challenge. This study strictly examines gene expression between high and low conception rate animals without influence from any exogenous metabolites, either dietary or pharmaceutical. Secondly, tissue sampling occurred during an estrous cycle where no embryo was present. Lastly, the study employed nulliparous heifers where the likelihood of uterine infection is low, as was demonstrated by the lack of clinical evidence of metritis, endometritis, pyometra or metaplasia across all heifers examined.

In total 55 DEG featured in inflammatory linked pathways. It is clear from the high proportion of DEG that inflammation is a central theme in estrous cycle and uterine sub-fertility physiology. *IL-33*, a cytokine which influences the production of other pro-inflammatory cytokines *IL-5*, *IL-13* and chemokine *GM-CSF*[113] was more highly expressed in LF animals. In addition, *IL-33* regulates transcription of endothelial cells in inflamed rheumatic tissues [114]. As mentioned previously, cell plasticity is altered in a state of chronic inflammation or trauma. Inflammation due to up-regulated *IL-33* could be altering the constitution of the endometrium in the LF animals, and thus impeding embryo implantation. Hence, low conception rates could be directly linked to inflammation induced, altered cellular plasticity, in uterine endometrial tissues.

### Metabolic exchange

Similar to Forde et al. [33], Bauersachs et al. [29] and Salilew-Wondim et al. [31], genes coding metabolite transporters, specifically the solute carrier (SLC) family members were found to be differentially expressed between HF and LF animals. The five SLC genes identified were; *SLC1A3*, *SLC17A5*, *SLC25A12*, *SLC25A24*, *SLC45A2*. The most abundantly expressed gene of the entire DEG list, *SLC45A2*, was 8-fold more highly expressed in the uterus of LF relative to HF heifers. As the name suggests SLC genes are involved in the transfer of solutes across the cell membrane, particularly amino acids [115–117]. Amino acids are fundamental for the normal growth and development of the early embryo, acting as precursors of nucleic acids and proteins, osmolytes and signaling molecules. Concentrations of amino acids in oviductal and uterine fluid during the estrous cycle have been reported to modulate with stage of cycle, systemic progesterone environment and differ compared with plasma, demonstrating their active transport in these tissues [118–121]. The endometrium functions as a secretory layer, suggesting the importance of metabolite exchange in this specific tissue. Animals with less efficient metabolic exchange in the uterus may be unable to sustain embryo development during early pregnancy, and thus be experiencing recurring early embryo loss.

Microarray analysis was carried out on endometrial tissue, an amalgam of varying cell types. Examining tissue mRNA gene expression provides an insight into the genetic regulation of multiple cell types from the host. It was essential to use RNA from all endometrial cell types as it is not apparent, as of yet, whether or which individual endometrial cell types are contributing to low conception rates in cattle. Investigations into the types and locations of contributing cell types via *in situ* hybridisation or immunofluorescence would assist in the development of proposed hypotheses.

## Conclusion

Global endometrial gene expression profiles during the mid-luteal phase of the estrous cycle, in HF and LF heifers was investigated, and the most significant biological pathways likely to be involved in uterine function and embryo survival identified. The new knowledge generated offers substantial insight into some of the molecular mechanisms underlying uterine endometrial function and uterine mediated low-fertility, during the early to mid luteal phase of the estrous cycle in cattle. Furthermore, expression analysis provides invaluable data on key differentially expressed genes which may be selected for future SNP discovery analysis which following validation may be used as genetic markers for fertility and incorporated into breeding programmes.

### Availability of supporting data

The data sets supporting the results of this article are available in the NCBI’s Gene Expression Omnibus repository, GSE29853 http://www.ncbi.nlm.nih.gov/geo/query/acc.cgi?acc=GSE29853.

## Electronic supplementary material

Additional file 1: Figure S1: Heatmap and dendogram following hierarchical clustering of differentially expressed genes. L: low fertility, H: high fertility heifers. (PDF 331 KB)

Additional file 2: Table S1: Up-regulated DEG (P < 0.05): Entrez ID, Symbol, Entrez Gene Name, Fold Change. **Table S2.** Down-regulated DEG (P < 0.05): Entrez ID, Symbol, Entrez Gene Name, Fold Change. **Table S3.** Genes validated between RT-qPCR and microarray methodologies, including Fold changes, P-values and correlation coefficients. (DOC 290 KB)
