# Supplementary figures and images for: Global gene expression in endometrium of high and low fertility heifers during the mid-luteal phase of the estrous cycle
Source: BMC Genomics. 2014 Mar 26;15(1):234. doi: 10.1186/1471-2164-15-234 (PMC3986929; doi:10.1186/1471-2164-15-234)

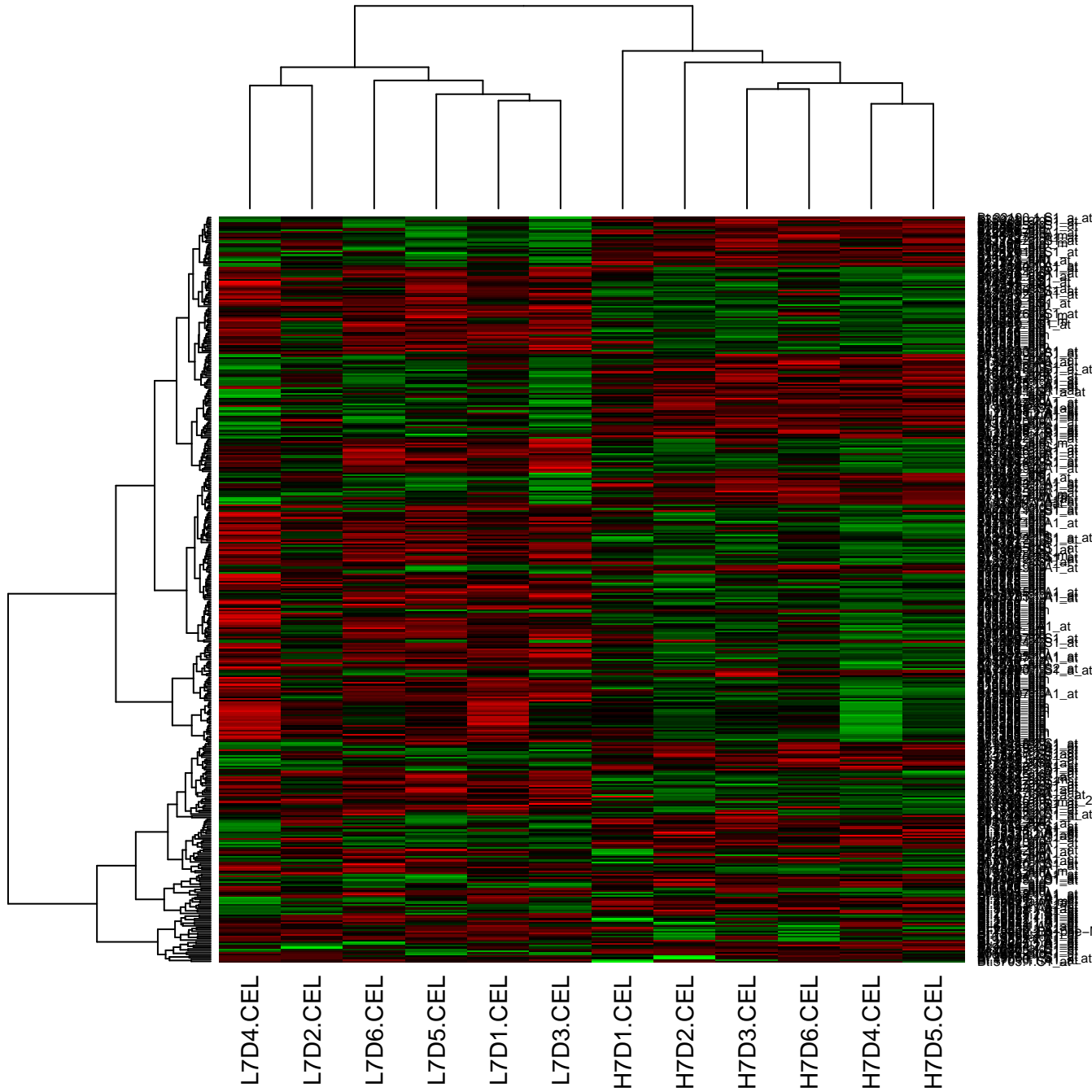

Supplement: Supplementary file 1 — Additional file 1: Figure S1: Heatmap and dendogram following hierarchical clustering of differentially expressed genes. L: low fertility, H: high fertility heifers. (PDF 331 KB) [file 12864_2013_7043_MOESM1_ESM.pdf]
