# Supplementary material for: Global gene expression in endometrium of high and low fertility heifers during the mid-luteal phase of the estrous cycle
Source: BMC Genomics. 2014 Mar 26;15(1):234. doi: 10.1186/1471-2164-15-234 (PMC3986929; doi:10.1186/1471-2164-15-234)
Supplement: Supplementary file 2 — Additional file 2: Table S1: Up-regulated DEG (P < 0.05): Entrez ID, Symbol, Entrez Gene Name, Fold Change. Table S2. Down-regulated DEG (P < 0.05): Entrez ID, Symbol, Entrez Gene Name, Fold Change. Table S3. Genes validated between RT-qPCR and microarray methodologies, including Fold changes, P-values and correlation coefficients. (DOC 290 KB) [file 12864_2013_7043_MOESM2_ESM.doc]

**Additional Table S1** Up-regulated DEG (*P* < 0.05): Entrez ID, Symbol, Entrez Gene Name, Fold Change

| **Entrez Gene ID** | **Symbol** | **Entrez Gene Name** | **FC** |
| --- | --- | --- | --- |
| 511082 | *ACAT1* | Acetyl-CoA acetyltransferase 1 | 1.140 |
| 504870 | *ACOT13* | Acyl-CoA thioesterase 13 | 1.152 |
| 539689 | *ALX1* | ALX homeobox 1 | 2.999 |
| 516065 | *ANKRD6* | Ankyrin repeat domain 6 | 1.151 |
| 613745 | *ARHGDIG* | Rho GDP dissociation inhibitor (GDI) gamma | 1.482 |
| 528191 | *BBS5* | Bardet-Biedl syndrome 5 | 1.148 |
| 614490 | *C11orf52* | Chromosome 11 open reading frame 52 | 1.462 |
| 525479 | *C20orf160* | Chromosome 20 open reading frame 160 | 1.419 |
| 508357 | *C9orf102* | Chromosome 9 open reading frame 102 | 1.169 |
| 327667 | *CACNB2* | Calcium channel, voltage-dependent, beta 2 subunit | 1.323 |
| 531682 | *CAT* | Catalase | 1.123 |
| 533020 | *CBR4* | Carbonyl reductase 4 | 1.165 |
| 782186 | *CD58* | CD58 molecule | 1.243 |
| 281139 | *CELA1* | Chymotrypsin-like elastase family, member 1 | 2.022 |
| 533443 | *CNOT8* | CCR4-NOT transcription complex, subunit 8 | 1.197 |
| 512761 | *CRLS1* | Gardiolipin synthase 1 | 1.189 |
| 613667 | *CXCL2* | Chemokine (C-X-C motif) ligand 2 | 1.392 |
| 281212 | *CXCL2* | Chemokine (C-X-C motif) ligand 2 | 1.545 |
| 530642 | *DCK* | Deoxycytidine kinase | 1.194 |
| 522248 | *DONSON* | Downstream neighbor of SON | 1.226 |
| 281128 | *DSC2* | Desmocollin 2 | 1.255 |
| 528046 | *EIF2C4* | Eukaryotic translation initiation factor 2C, 4 | 1.112 |
| 768074 | *ETFDH* | Electron-transferring-flavoprotein dehydrogenase | 1.190 |
| 615773 | *FAM119A* | Family with sequence similarity 119, member A | 1.336 |
| 516522 | *FRRS1* | Ferric-chelate reductase 1 | 1.291 |
| 781914 | *FTSJD1* | FtsJ methyltransferase domain containing 1 | 1.113 |
| 618405 | *GADD45B* | Growth arrest and DNA-damage-inducible, beta | 1.250 |
| 506903 | *GALNT6* | UDP-N-acetyl-alpha-D-galactosamine:polypeptide N-acetylgalactosaminyltransferase 6 (GalNAc-T6) | 6.648 |
| 281199 | *GLUL* | Glutamate-ammonia ligase | 1.221 |
| 509296 | *GSDMB* | Gasdermin B | 2.639 |
| 510504 | *GYLTL1B* | Glycosyltransferase-like 1B | 1.141 |
| 533336 | *HERC4* | Hect domain and RLD 4 | 1.074 |
| 534001 | *HNRNPH2* | Heterogeneous nuclear ribonucleoprotein H2 (H') | 1.219 |
| 507054 | *IL33* | Interleukin 33 | 1.348 |
| 538461 | *KBTBD6* | Kelch repeat and BTB (POZ) domain containing 6 | 1.885 |
| 784675 | *KIAA1462* | KIAA1462 | 1.164 |
| 535021 | *LACTB2* | Lactamase, beta 2 | 1.189 |
| 281285 | *LRAT* | Lecithin retinol acyltransferase (phosphatidylcholine--retinol O-acyltransferase) | 1.467 |
| 524868 | *MED13* | Mediator complex subunit 13 | 1.110 |
| 613844 | *METTL7A* | Methyltransferase like 7A | 1.222 |
| 615506 | *MOSC2* | MOCO sulphurase C-terminal domain containing 2 | 1.122 |
| 504457 | *NSF* | N-ethylmaleimide-sensitive factor | 1.094 |
| 537455 | *NTN4* | Netrin 4 | 1.215 |
| 527318 | *PACRGL* | PARK2 co-regulated-like | 1.263 |
| 615359 | *PARP4* | Poly (ADP-ribose) polymerase family, member 4 | 1.081 |
| 515902 | *PCCB* | Propionyl CoA carboxylase, beta polypeptide | 1.136 |
| 521261 | *PCMTD1* | Protein-L-isoaspartate (D-aspartate) O-methyltransferase domain containing 1 | 1.104 |
| 281975 | *PDE6C* | Phosphodiesterase 6C, cGMP-specific, cone, alpha prime | 1.992 |
| 524913 | *PIBF1* | Progesterone immunomodulatory binding factor 1 | 1.125 |
| 531199 | *PLIN5* | Perilipin 5 | 1.910 |
| 281992 | *PPARA* | Peroxisome proliferator-activated receptor alpha | 1.372 |
| 282030 | *RAB3B* | RAB3B, member RAS oncogene family | 1.852 |
| 504785 | *RABEP1* | Rabaptin, RAB GTPase binding effector protein 1 | 1.136 |
| 616969 | *RGNEF* | 190 kDa guanine nucleotide exchange factor | 1.157 |
| 533646 | *RIT1* | Ras-like without CAAX 1 | 1.114 |
| 535869 | *RNF128* | Ring finger protein 128 | 1.226 |
| 524166 | *RNF144B* | Ring finger protein 144B | 1.255 |
| 538571 | *RSPRY1* | Ring finger and SPRY domain containing 1 | 1.163 |
| 530164 | *SLC17A5* | Solute carrier family 17 (anion/sugar transporter), member 5 | 1.145 |
| 534742 | *SLC25A24* | Solute carrier family 25 (mitochondrial carrier; phosphate carrier), member 24 | 1.216 |
| 538746 | *SLC45A2* | Solute carrier family 45, member 2 | 8.003 |
| 507462 | *SNX16* | Sorting nexin 16 | 1.148 |
| 534321 | *SOX6* | SRY (sex determining region Y)-box 6 | 1.236 |
| 614612 | *SRD5A1* | Steroid-5-alpha-reductase, alpha polypeptide 1 (3-oxo-5 alpha-steroid delta 4-dehydrogenase alpha 1) | 1.130 |
| 505945 | *SRPK2* | SRSF protein kinase 2 | 1.110 |
| 540203 | *TAB2* | TGF-beta activated kinase 1/MAP3K7 binding protein 2 | 1.086 |
| 539324 | *TMEM30B* | Transmembrane protein 30B | 1.153 |
| 515078 | *TMEM62* | Transmembrane protein 62 | 1.131 |
| 507934 | *TOB2* | Transducer of ERBB2, 2 | 1.116 |
| 515682 | *TP53I11* | Tumor protein p53 inducible protein 11 | 1.390 |
| 506991 | *TXNDC12* | Thioredoxin domain containing 12 (endoplasmic reticulum) | 1.147 |
| 281563 | *UCP3* | Uncoupling protein 3 (mitochondrial, proton carrier) | 1.906 |
| 780856 | *YPEL5* | Yippee-like 5 (Drosophila) | 1.098 |

**Additional Table S2.** Down-regulated DEG (*P* < 0.05): Entrez ID, Symbol, Entrez Gene Name, Fold Change

| **Entrez Gene ID** | **Symbol** | **Entrez Gene Name** | **FC** |
| --- | --- | --- | --- |
| 515610 | *ACTA2* | Actin, alpha 2, smooth muscle, aorta | 1.679 |
| 280979 | *ACTB* | Actin, beta | 1.160 |
| 533219 | *ACTC1* | Actin, alpha, cardiac muscle 1 | 1.505 |
| 510708 | *ADCY2* | Adenylate cyclase 2 (brain) | 1.498 |
| 535603 | *ADCY3* | Adenylate cyclase 3 | 1.216 |
| 532836 | *AHCYL2* | Adenosylhomocysteinase-like 2 | 1.185 |
| 533620 | *ANKRD26* | Ankyrin repeat domain 26 | 1.505 |
| 327685 | *ANXA6* | Annexin A6 | 1.232 |
| 514666 | *APEH* | N-acylaminoacyl-peptide hydrolase | 1.063 |
| 511220 | *ARHGEF11* | Rho guanine nucleotide exchange factor (GEF) 11 | 1.256 |
| 506075 | *ARHGEF25* | Rho guanine nucleotide exchange factor (GEF) 25 | 1.257 |
| 509596 | *ATP13A5* | ATPase type 13A5 | 1.196 |
| 782633 | *BAG3* | BCL2-associated athanogene 3 | 1.154 |
| 514945 | *BMS1* | BMS1 homolog, ribosome assembly protein (yeast) | 1.121 |
| 512018 | *BOC* | Boc homolog (mouse) | 1.272 |
| 614800 | *C19orf53* | Chromosome 19 open reading frame 53 | 1.152 |
| 506413 | *C1QTNF6* | C1q and tumor necrosis factor related protein 6 | 1.316 |
| 540879 | *C21orf7* | Chromosome 21 open reading frame 7 | 1.189 |
| 509253 | *C7orf30* | Chromosome 7 open reading frame 30 | 1.291 |
| 506598 | *CARS2* | Cysteinyl-tRNA synthetase 2, mitochondrial (putative) | 1.150 |
| 508712 | *CCBL2* | Cysteine conjugate-beta lyase 2 | 1.119 |
| 540069 | *CIAO1* | Cytosolic iron-sulfur protein assembly 1 | 1.067 |
| 509965 | *CMPK1* | Cytidine monophosphate (UMP-CMP) kinase 1, cytosolic | 1.061 |
| 507010 | *COL16A1* | Collagen, type XVI, alpha 1 | 1.283 |
| 282188 | *COL1A2* | Collagen, type I, alpha 2 | 1.185 |
| 523526 | *COL4A6* | Collagen, type IV, alpha 6 | 1.329 |
| 281706 | *COPB2* | Coatomer protein complex, subunit beta 2 (beta prime) | 1.083 |
| 617620 | *CORO2A* | Coronin, actin binding protein, 2A | 1.501 |
| 281090 | *COX4I1* | Cytochrome c oxidase subunit IV isoform 1 | 1.107 |
| 615329 | *CSRP1* | Cysteine and glycine-rich protein 1 | 1.193 |
| 539381 | *CSRP2* | Cysteine and glycine-rich protein 2 | 1.206 |
| 281108 | *CTSL2* | Cathepsin L2 | 1.122 |
| 516091 | *CUEDC2* | CUE domain containing 2 | 1.101 |
| 616066 | *DAP* | Death-associated protein | 1.125 |
| 507590 | *DBNDD2* | Dysbindin (dystrobrevin binding protein 1) domain containing 2 | 1.271 |
| 509796 | *DPYSL3* | Dihydropyrimidinase-like 3 | 1.154 |
| 281138 | *EEF2* | Eukaryotic translation elongation factor 2 | 1.120 |
| 532568 | *ELAC1* | ElaC homolog 1 (E. coli) | 1.105 |
| 617293 | *ELOVL5* | ELOVL family member 5, elongation of long chain fatty acids (FEN1/Elo2, SUR4/Elo3-like, yeast) | 1.283 |
| 535273 | *EMP3* | Epithelial membrane protein 3 | 1.147 |
| 615535 | *ENPP1* | Ectonucleotide pyrophosphatase/phosphodiesterase 1 | 1.162 |
| 508901 | *ERH* | Enhancer of rudimentary homolog (Drosophila) | 1.164 |
| 618107 | *FAM167A* | Family with sequence similarity 167, member A | 1.495 |
| 281152 | *FASN* | Fatty acid synthase | 1.126 |
| 282847 | *FAU* | Finkel-Biskis-Reilly murine sarcoma virus (FBR-MuSV) ubiquitously expressed | 1.136 |
| 616981 | *FERMT2* | Fermitin family member 2 | 1.103 |
| 510008 | *FHL2* | Four and a half LIM domains 2 | 1.227 |
| 504795 | *FHL3* | Four and a half LIM domains 3 | 1.191 |
| 535310 | *FKBP10* | FK506 binding protein 10, 65 kDa | 1.209 |
| 509422 | *FKBP7* | FK506 binding protein 7 | 1.140 |
| 538717 | *FNBP1* | Formin binding protein 1 | 1.266 |
| 505865 | *FOLH1* | Folate hydrolase (prostate-specific membrane antigen) 1 | 1.267 |
| 327681 | *FST* | Follistatin | 1.385 |
| 280796 | *FUS* | Fused in sarcoma | 1.110 |
| 616218 | *GALNT2* | UDP-N-acetyl-alpha-D-galactosamine:polypeptide N-acetylgalactosaminyltransferase 2 (GalNAc-T2) | 1.165 |
| 511617 | *GIMAP5* | GTPase, IMAP family member 5 | 1.331 |
| 281193 | *GJA1* | Gap junction protein, alpha 1, 43kDa | 1.262 |
| 785371 | *GLI3* | GLI family zinc finger 3 | 1.293 |
| 616809 | *GOLGA7* | Golgin A7 | 1.068 |
| 505093 | *Hcg 2023776* | Heterogeneous nuclear ribonucleoprotein A1 pseudogene 8 | 1.142 |
| 507723 | *HMG20B* | High-mobility group 20B | 1.139 |
| 282691 | *HMGB1* | High-mobility group box 1 | 1.121 |
| 516099 | *HSPB1* | Heat shock 27kDa protein 1 | 1.132 |
| 782019 | *IER5L* | Immediate early response 5-like | 1.128 |
| 512913 | *IFI6* | Interferon, alpha-inducible protein 6 | 1.347 |
| 536731 | *IFT122* | Intraflagellar transport 122 homolog (Chlamydomonas) | 1.118 |
| 617147 | *IFT27* | Intraflagellar transport 27 homolog (Chlamydomonas) | 1.122 |
| 522155 | *IL6ST* | Interleukin 6 signal transducer (gp130, oncostatin M receptor) | 1.131 |
| 614936 | *ILF3* | Interleukin enhancer binding factor 3, 90kDa | 1.136 |
| 511969 | *IMPDH2* | IMP (inosine 5'-monophosphate) dehydrogenase 2 | 1.091 |
| 509181 | *JKAMP* | JNK1/MAPK8-associated membrane protein | 1.115 |
| 407176 | *KCNMB1* | Potassium large conductance calcium-activated channel, subfamily M, beta member 1 | 1.782 |
| 513714 | *KRR1* | KRR1, small subunit (SSU) processome component, homolog (yeast) | 1.088 |
| 505690 | *LARP1* | La ribonucleoprotein domain family, member 1 | 1.178 |
| 326598 | *LGALS1* | Lectin, galactoside-binding, soluble, 1 | 1.204 |
| 786492 | *LGALS3* | Lectin, galactoside-binding, soluble, 3 | 1.209 |
| 281278 | *LPHN2* | Latrophilin 2 | 1.228 |
| 781032 | *LTBP1* | Latent transforming growth factor beta binding protein 1 | 1.384 |
| 510977 | *LY6E* | Lymphocyte antigen 6 complex, locus E | 1.138 |
| 512562 | *MAGED1* | Melanoma antigen family D, 1 | 1.231 |
| 613548 | *MARCKS* | Myristoylated alanine-rich protein kinase C substrate | 2.106 |
| 781397 | *MARVELD1* | MARVEL domain containing 1 | 1.164 |
| 525389 | *MED27* | Mediator complex subunit 27 | 1.158 |
| 514610 | *MESDC2* | Mesoderm development candidate 2 | 1.125 |
| 404180 | *MEST* | Mesoderm specific transcript homolog (mouse) | 1.299 |
| 533203 | *MMP19* | Matrix metallopeptidase 19 | 1.240 |
| 506657 | *MRPL55* | Mitochondrial ribosomal protein L55 | 1.257 |
| 281918 | *MRVI1* | Murine retrovirus integration site 1 homolog | 1.253 |
| 338037 | *MYLK* | Myosin light chain kinase | 1.346 |
| 522383 | *NES* | Nestin | 1.252 |
| 506584 | *NMB* | Neuromedin B | 1.781 |
| 615447 | *NME1-NME2* | NME1-NME2 readthrough | 1.217 |
| 281356 | *NPPC* | Natriuretic peptide C | 1.858 |
| 281949 | *NR0B1* | Nuclear receptor subfamily 0, group B, member 1 | 1.377 |
| 281365 | *ODC1* | Ornithine decarboxylase 1 | 1.143 |
| 505318 | *OLFML3* | Olfactomedin-like 3 | 1.181 |
| 519409 | *ORC5* | Origin recognition complex, subunit 5 | 1.303 |
| 281963 | *P2RY1* | Purinergic receptor P2Y, G-protein coupled, 1 | 1.488 |
| 404170 | *PAICS* | Phosphoribosylaminoimidazole carboxylase, phosphoribosylaminoimidazole succinocarboxamide synthetase | 1.203 |
| 511268 | *PARK7* | Parkinson disease (autosomal recessive, early onset) 7 | 1.116 |
| 617094 | *PGM5* | Phosphoglucomutase 5 | 1.354 |
| 540135 | *PHLDA1* | Pleckstrin homology-like domain, family A, member 1 | 1.219 |
| 786612 | *PJA1* | Praja ring finger 1 | 1.214 |
| 537070 | *PMM1* | Phosphomannomutase 1 | 1.117 |
| 505599 | *POLR2H* | Polymerase (RNA) II (DNA directed) polypeptide H | 1.098 |
| 513428 | *PPIH* | Peptidylprolyl isomerase H (cyclophilin H) | 1.107 |
| 282875 | *PRKRA* | Protein kinase, interferon-inducible double stranded RNA dependent activator | 1.175 |
| 520388 | *PRMT1* | Protein arginine methyltransferase 1 | 1.093 |
| 282328 | *PSMB10* | Proteasome (prosome, macropain) subunit, beta type, 10 | 1.165 |
| 513461 | *PSMD11* | Proteasome (prosome, macropain) 26S subunit, non-ATPase, 11 | 1.086 |
| 539784 | *PSMD2* | Proteasome (prosome, macropain) 26S subunit, non-ATPase, 2 | 1.114 |
| 282021 | *PTGIS* | Prostaglandin I2 (prostacyclin) synthase | 1.166 |
| 509657 | *PTPRK* | Protein tyrosine phosphatase, receptor type, K | 1.097 |
| 493722 | *QKI* | Quaking homolog, KH domain RNA binding (mouse) | 1.154 |
| 615804 | *RAB12* | RAB12, member RAS oncogene family | 1.078 |
| 282028 | *RAB34* | RAB34, member RAS oncogene family | 1.158 |
| 535393 | *RAB3GAP1* | RAB3 GTPase activating protein subunit 1 (catalytic) | 1.114 |
| 508990 | *RARRES2* | Retinoic acid receptor responder (tazarotene induced) 2 | 1.261 |
| 537402 | *RBBP7* | Retinoblastoma binding protein 7 | 1.174 |
| 525636 | *RCAN2* | Regulator of calcineurin 2 | 1.153 |
| 522073 | *RCN3* | Reticulocalbin 3, EF-hand calcium binding domain | 1.221 |
| 617543 | *REEP5* | Receptor accessory protein 5 | 1.146 |
| 781044 | *RHOU* | Ras homolog gene family, member U | 1.224 |
| 505618 | *RNASEH2C* | Ribonuclease H2, subunit C | 1.104 |
| 504876 | *RPL23* | Ribosomal protein L23 | 1.148 |
| 337890 | *RPL26* | Ribosomal protein L26 | 1.120 |
| 507270 | *RPL29* | Ribosomal protein L29 | 1.131 |
| 533285 | *RPLP1* | Ribosomal protein, large, P1 | 1.156 |
| 286853 | *RPLP2* | Ribosomal protein, large, P2 | 1.121 |
| 326588 | *RPS3* | Ribosomal protein S3 | 1.168 |
| 506229 | *RPS5* | Ribosomal protein S5 | 1.153 |
| 511675 | *RSPO1* | R-spondin homolog (Xenopus laevis) | 1.717 |
| 508224 | *SCG5* | Secretogranin V (7B2 protein) | 1.286 |
| 513180 | *SDCCAG3* | Serologically defined colon cancer antigen 3 | 1.207 |
| 506636 | *SEMA5A* | Sema domain, seven thrombospondin repeats (type 1 and type 1-like), transmembrane domain (TM) and short cytoplasmic domain, (semaphorin) 5A | 1.200 |
| 510850 | *SERPINH1* | Serpin peptidase inhibitor, clade H (heat shock protein 47), member 1, (collagen binding protein 1) | 1.274 |
| 282068 | *SFRP1* | Secreted frizzled-related protein 1 | 1.481 |
| 535511 | *SH3D19* | SH3 domain containing 19 | 1.157 |
| 282354 | *SLC1A3* | Solute carrier family 1 (glial high affinity glutamate transporter), member 3 | 1.730 |
| 539494 | *SLC25A12* | Solute carrier family 25 (mitochondrial carrier, Aralar), member 12 | 1.117 |
| 615490 | *SMOC2* | SPARC related modular calcium binding 2 | 1.250 |
| 530618 | *SPOP* | Speckle-type POZ protein | 1.284 |
| 614029 | *SREK1IP1* | SREK1-interacting protein 1 | 1.114 |
| 444859 | *ST3GAL3* | ST3 beta-galactoside alpha-2,3-sialyltransferase 3 | 1.189 |
| 534816 | *STIM1* | Stromal interaction molecule 1 | 1.186 |
| 282485 | *SULT1A1* | Sulfotransferase family, cytosolic, 1A, phenol-preferring, member 1 | 1.130 |
| 509837 | *SUOX* | Sulfite oxidase | 1.201 |
| 280936 | *SV2A* | Synaptic vesicle glycoprotein 2A | 1.221 |
| 509039 | *TCF12* | Transcription factor 12 | 1.088 |
| 515834 | *TGFB1I1* | Transforming growth factor beta 1 induced transcript 1 | 1.263 |
| 539627 | *TNIK* | TRAF2 and NCK interacting kinase | 1.195 |
| 505366 | *WFDC1* | WAP four-disulfide core domain 1 | 1.284 |
| 504523 | *ZNF259* | Zinc finger protein 259 | 1.211 |
| 512477 | *ZNF48* | Zinc finger protein 48 | 1.136 |

**Additional Table S3. Genes validated between RT-qPCR and microarray methodologies, including Fold changes, *P*-values and correlation coefficients (n=12).**

|  | **Microarray** | | **RT-qPCR** | | **Correlation** | |
| --- | --- | --- | --- | --- | --- | --- |
| **Gene** | **FC** | ***P-*Value** | **FC** | ***P-*Value** | **R** | ***P-*Value** |
| *ACTA2* | -1.74 | 0.007 | -2.24 | 0.037 | 0.91 | <0.001 |
| *CELA1* | +2.02 | 0.013 | +2.49 | 0.043 | 0.88 | 0.001 |
| *DONSON* | 1.23 | 0.008 | +1.24 | 0.005 | 0.89 | 0.002 |
| *FST* | -1.38 | <0.001 | -1.32 | 0.648 | 0.35 | 0.262 |
| *GALNT6* | +6.65 | 0.001 | +2.01 | 0.011 | 0.94 | <0.001 |
| *GJA1* | -1.26 | 0.008 | -1.46 | 0.043 | 0.76 | 0.004 |
| *IL-33* | +1.35 | 0.021 | +1.99 | 0.020 | 0.03 | 0.945 |
| *MMP19* | -1.24 | 0.004 | -1.68 | 0.003 | 0.94 | <0.001 |
| *MOSC2* | +1.12 | 0.022 | +1.25 | 0.027 | 0.80 | 0.003 |
| *NMB* | -1.78 | 0.007 | -2.60 | 0.022 | 0.91 | <0.001 |
| *NPPC* | -1.86 | 0.004 | -1.53 | 0.047 | 0.94 | <0.001 |
| *PPARA* | +1.37 | 0.006 | +1.05 | 0.291 | 0.28 | 0.386 |
| *RAB3B* | +1.85 | 0.024 | +1.52 | 0.019 | 0.91 | <0.001 |
| *SFRP1* | -1.48 | 0.000 | -2.96 | 0.027 | 0.61 | 0.059 |
| *SLC1A3* | -1.73 | 0.009 | -2.08 | 0.020 | 0.79 | 0.002 |
| *SLC45A2* | +8.00 | 0.000 | +2.33 | 0.034 | 0.78 | 0.008 |
| *TGFB1I1* | -1.26 | 0.007 | -2.25 | 0.003 | 0.87 | <0.001 |
| *APEH* | -1.06 | 0.034 | -1.01 | 0.908 | -0.16 | 0.622 |
